# High NANOG expression correlates with worse patients’ survival in esophageal adenocarcinoma

**DOI:** 10.1186/s12885-023-11146-0

**Published:** 2023-07-17

**Authors:** Karl Knipper, Alexander I. Damanakis, Su Ir Lyu, Adrian Georg Simon, Isabell Wahler, Christiane J. Bruns, Wolfgang Schröder, Thomas Schmidt, Alexander Quaas

**Affiliations:** 1 Faculty of Medicine and University Hospital of Cologne, Department of General, Visceral and Cancer Surgery, University of Cologne, Cologne, Germany; 2 Faculty of Medicine and University Hospital of Cologne, Institute of Pathology, University of Cologne, Cologne, Germany

**Keywords:** Esophageal adenocarcinoma, NANOG, Multimodal therapy, Personalized medicine

## Abstract

**Background:**

Patients diagnosed with esophageal cancer demonstrate a low overall survival even despite the established multimodal therapy as the current standard of care. Therefore, further biomarkers for patients with high-risk and additional therapy options are needed. NANOG is a transcription factor, which can be found in stem cells and is known to support tumorigenesis.

**Methods:**

Six hundred sixty patients with esophageal adenocarcinoma, who were operated at the University of Cologne with a curative intent, were included. Immunohistochemical stainings for NANOG were performed. The study population was divided into NANOG-positive and -negative subgroups.

**Results:**

Positive NANOG expression correlates significantly with worse overall survival (*p* = 0.002) and could be confirmed as an independent risk factor for worse patient survival in multivariate analysis (HR = 1.40, 95%CI = 1.09–1.80, *p* = 0.006). This effect could be detected in the subgroup of primarily operated patients, but not in patients after neoadjuvant therapy.

**Conclusions:**

We describe a NANOG-positive subgroup of patients with esophageal cancer, who exhibit worse overall survival in a large patient cohort. This discovery suggests the potential use of NANOG as a biomarker for both intensified therapy and stricter follow-up regimes. Additionally, NANOG-positive stem cell-like cancer cells could be used as a new antitumoral treatment target if validated in mechanistic and clinical studies.

**Supplementary Information:**

The online version contains supplementary material available at 10.1186/s12885-023-11146-0.

## Background

Counted worldwide, estimated 572,034 new cases of esophageal cancer occurred in 2018 [1]. Especially in Western Europe, the incidence of esophageal adenocarcinoma is increasing despite decreasing exposure to the risk factors [2, 3]. Esophageal cancer metastases are linked to a significantly worse patient outcome [4]. The initiation of metastases has not been fully understood yet. One possible pathway is that metastases arise directly from the cancer stem cells, as they were shown to have a significantly higher migratory capacity and could be detected in the invasive front of pancreatic adenocarcinoma in mice [5]. Furthermore cancer stem cells are not only involved in metastases but also in the tumorigenesis [6]. Hence, these cells contribute to the overall advancement of the tumor. However, the role of the cancer stem cells seems to show divergence between different cancer entities [6].

NANOG is a homeodomain protein, which has a crucial transcriptional role in the differentiation of embryonic stem cells [7]. Increased NANOG expression levels lead to a propagation of undifferentiated stem cells [7]. NANOG is involved in a complex regulatory system. For instance, p53 suppresses NANOG expression and causes stem cell differentiation while STAT3 induces NANOG expression [8, 9]. NANOG promoted proliferation and invasion in esophageal squamous cell carcinoma cell lines. Furthermore, elevated NANOG expression levels were shown to inhibit apoptosis and decrease sensitivity to cisplatin [10]. NANOG overexpression could be observed as a factor for poorer patient survival in several tumor entities, for instance in gastric adenocarcinoma as well as in colorectal cancer [11, 12].

This study aims to elucidate the role of NANOG in esophageal adenocarcinoma.

## Methods

### Patients and tumor samples

This study includes patients with esophageal adenocarcinoma, who underwent surgical treatment between 1996 and 2019 at the University Hospital of Cologne with a curative intent. As no cancer cells were present to evaluate NANOG staining, patients with a pathological complete response after neoadjuvant therapy were excluded from the analysis. Clinicopathological data were collected prospectively and then analyzed retrospectively. The overall survival was defined as the time between surgery and either death or loss of follow-up. Patients who either passed away or were lost to follow-up within 30 days after surgery were excluded from the analysis. This study was performed in accordance with the principles of the Declaration of Helsinki. Approval was granted by the ethics committee of the University of Cologne. Informed consent was obtained from all of the individual participants included in the study.

1.2 mm thick tissue cylinders were punched out of the tumor samples with a semi-automated instrument and transferred into paraffin blocks as described before [13].

### Immunohistochemistry (IHC) and Analysis

Immunohistochemical stainings for NANOG and TP53 were performed using the Leica Bond-MAX automated system (Leica Biosystems, Wetzlar, Germany) (for details refer to Supplement Table 1). The staining intensity was determined semiquantitatively by an experienced pathologist (AQ). Tumors without expression of NANOG were classified as negative, tumors with low expression intensity, moderate intensity in ≤ 70% or strong intensity in ≤ 30% of tumor cells were classified as low positive, and tumors with moderate intensity in > 70% of tumor cells or strong intensity in > 30% were classified as high positive. NANOG expression patterns were generally grouped in either negative or positive NANOG expression. Additionally, we grouped NANOG expression patterns in negative, low, and high expression for subanalyses.

### RNA-Sequencing analysis of the TCGA cohort (The Cancer Genome Atlas)

The Broad Institute Firehose GDAC portal was used for retrieval of normalized RNA counts (Transcripts per Million (TPM), RNA-Sequencing by Expectation–Maximization normalization) and clinical data (https://gdac.broadinstitute.org/). This openly available patient cohort consists of 88 cases of esophageal adenocarcinoma treated with primary surgery solely. Histopathological data as well as survival data (overall survival) were available (Supp. Table 5). To account for potential artefacts and background noise during RNA sequencing, a NANOG mRNA expression cutoff of TPM = 5.0 was chosen for the dichotomization. For survival analysis, the cohort was subsequently dichotomized into tumors with a NANOG expression of TPM > 5.0 (NANOG expressed) and tumors with a NANOG expression TPM < 5.0 (NANOG not expressed).

### Statistical analysis

Statistical analyses were carried out with R and R Studio for Mac, Version 4.2.2. Continuous values were compared using the Wilcoxon rank sum test and categorical variables using Chi-square test, if not stated differently. Survival analyses were performed with Kaplan–Meier curves and analyzed using the log rank test. Univariate and multivariate cox regression analyses were done to detect correlations between patients' survival and the clinicopathologic characteristics. *P*-values below 0.05 were interpreted as significant.

## Results

Six hundred sixty patients with esophageal adenocarcinoma were included in this study. Surgeries were performed at the University Hospital of Cologne between 1996 and 2019. Detailed patient characteristics are shown in Table 1. 87% of the study population was male. The median age was 64 years. 432 (65%) patients received a (radio-)chemotherapy prior to resection. The majority of the included patients was diagnosed with local (y)pT3 cancer (64%). In 63% of all the included cases at least one lymph node metastasis could be detected ((y)pN +).

**Table 1:** Patients’ characteristics of the total population and NANOG negative and positive subgroup. UICC: Union for International Cancer Control. Bold print marks p-values below 0.05

**Characteristic**	**Total**	**NANOG negative**	**NANOG positive**	
	**n (%)**	**n (%)**	**n (%)**	
**No. of patients**	660 (100)	168 (100)	492 (100)	
**Sex**				0.900
Male	576 (87)	146 (87)	430 (87)	
Female	84 (13)	22 (13)	62 (13)	
**Median Age (years)**	64	66	63	
**(range)**	(56–71)	(57–72)	(55–71)	
**Neoadjuvant therapy**				**0.009**
No	228 (35)	72 (43)	156 (32)	
Yes	432 (65)	96 (57)	336 (68)	
**Neoadjuvant treatment regime**				0.832
CROSS	142 (33)	26 (27)	116 (35)	
FLOT	77 (18)	15 (16)	62 (18)	
Not specified	213 (49)	55 (57)	158 (47)	
**NANOG**				
Negative	168 (25)	168 (100)	0 (0)	
Positive	492 (75)	0 (0)	492 (100)	
**pT**				0.071
1	107 (16)	37 (22)	70 (14)	
2	107 (16)	24 (14)	83 (17)	
3	424 (64)	104 (62)	320 (65)	
4	22 (4)	3 (2)	19 (4)	
**pN**				0.081
0	246 (37)	76 (45)	170 (35)	
1	201 (30)	48 (29)	153 (31)	
2	103 (16)	22 (13)	81 (16)	
3	110 (17)	22 (13)	88 (18)	
**UICC**				0.082
I	77 (12)	27 (16)	50 (10)	
II	70 (11)	20 (12)	50 (10)	
III	300 (45)	77 (49)	223 (45)	
IV	213 (32)	44 (26)	169 (34)	

We divided our study population into a negative (*n* = 168) and a positive (*n* = 492) subgroup based on NANOG expression. Furthermore, we compared the clinicopathological data of both groups (Table 1). There is an accumulation of NANOG-positive EACs in the group of neoadjuvant-treated patients (*p* = 0.009). No significant differences between NANOG expression and prior treatment with FLOT compared with CROSS could be detected (*p* = 0.832).

**Fig. 1:**
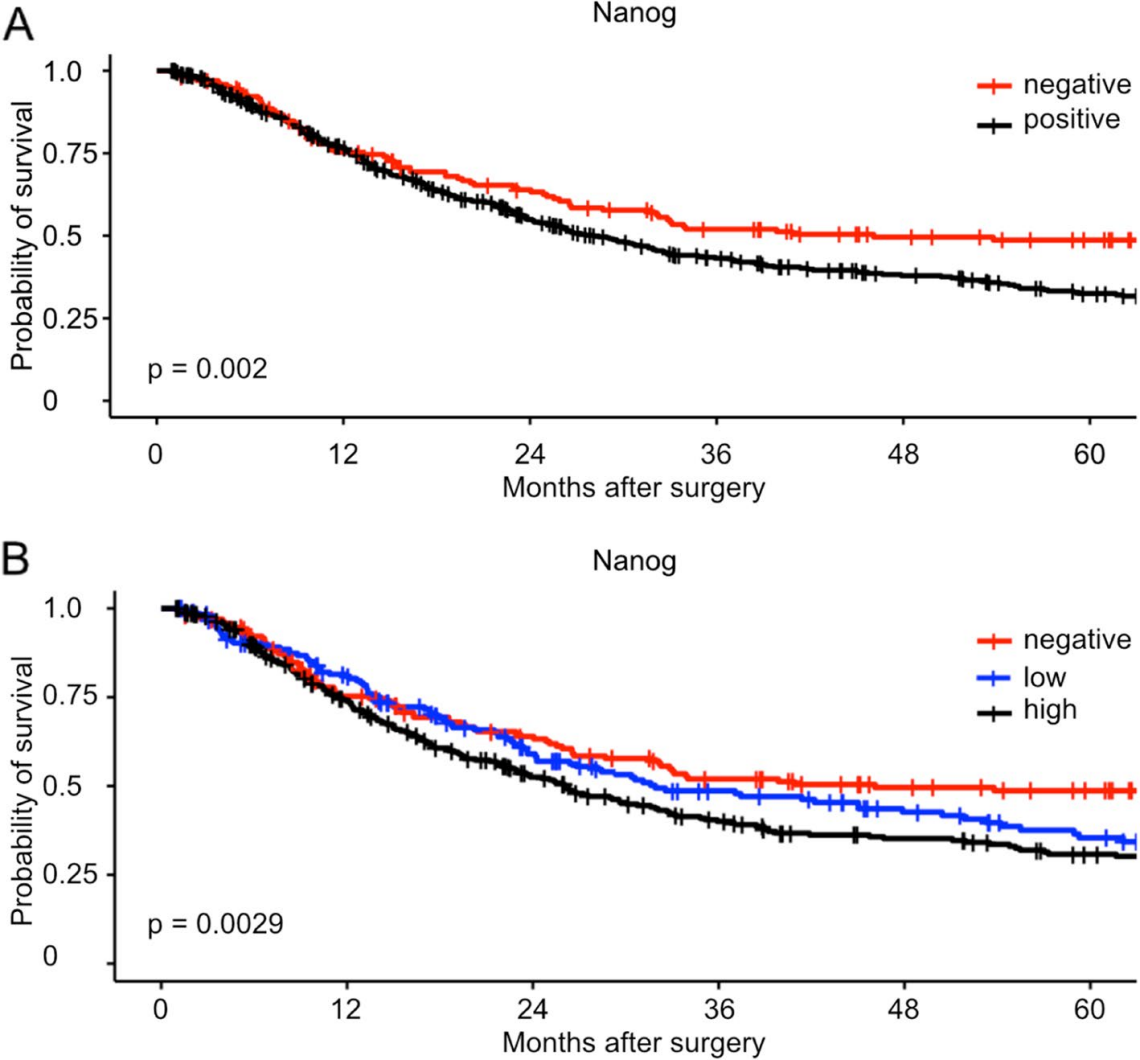
Analysis of overall survival depending on (**A**) negative or positive expression of NANOG (n(negative) = 168, n(positive) = 492, *p* = 0.002), and (**B**) negative, low, or high expression of NANOG (n(negative) = 168, n(low) = 183, n(high) = 309, *p* = 0.0029)

We analyzed the overall survival of patients based on their NANOG expression status. Patients with NANOG expression showed a significantly worse overall survival compared to those without (negative: median Follow-Up = 28.21 months, n = 168, positive: median Follow-Up = 21.36 months, *n* = 492, *p* = 0.002, Fig. 1A). We also performed survival analyses when dividing the patient cohort into negative, low, and high NANOG expression groups. In this analysis, both low and high NANOG expression patterns were associated with significantly poorer survival rates (negative: *n* = 168, low: *n* = 183, high: *n* = 309, *p* = 0.0029, Fig. 1 B).

Following this, univariate analyses for all clinicopathological values and NANOG staining were performed, which showed that higher age, neoadjuvant therapy, higher pN-, higher pT-, higher UICC-stages and positive NANOG expression were significantly correlated with worse overall survival (Supp. Table 2).

Additionally, we performed multivariate cox regression analyses to detect any interdependencies. Positive NANOG expression showed to be a significant risk factor for poorer overall survival in esophageal adenocarcinoma (HR = 1.40, 95% CI = 1.09–1.80, p = 0.006, Table 2). Moreover, male sex, higher age, and a higher pT as well as pN stage are independent risk factors for worse patient survival (sex: HR = 1.45, 95% CI = 1.04–2.03, *p* = 0.020; age: HR = 1.02, 95% CI = 1.01–1.03, *p* < 0.001; pT 4 vs 1: HR = 2.44, 95% CI = 1.24–4.79, *p* < 0.001; pN 3 vs 0: HR = 3.89, 95% CI = 2.86–5.29, *p* < 0.001, Table 2).

**Table 2:** Multivariate cox regression of the total population. Bold print marks p-values below 0.05

Characteristic	Borders	Hazard Ratio	95% confidence interval	*p*—value
**Sex**	male vs female	1.45	1.04—2.03	**0.020**
**Age**		1.02	1.01—1.03	** < 0.001**
**Neoadjuvant therapy**	yes vs no	1.07	0.814—1.37	0.600
**pT**				** < 0.001**
	2 vs 1	1.75	1.14—2.69	
	3 vs 1	2.02	1.38—2.94	
	4 vs 1	2.44	1.24—4.79	
**pN**				** < 0.001**
	1 vs 0	1.95	1.48—2.56	
	2 vs 0	2.35	1.70—3.24	
	3 vs 0	3.89	2.86—5.29	
**NANOG**	positive vs negative	1.40	1.09—1.80	**0.006**

Since we could observe significantly higher NANOG expression in the neoadjuvant treated patients, we conducted subgroup analyses for patients who received neoadjuvant therapy and those who underwent primary surgery. Detailed patient characteristics of these two subgroups are shown in Supplement Table 3. Neoadjuvant treated patients showed significantly higher UICC- or pT-stadium and significantly more NANOG-positive tumors (Supp. Table 3).

**Fig. 2:**
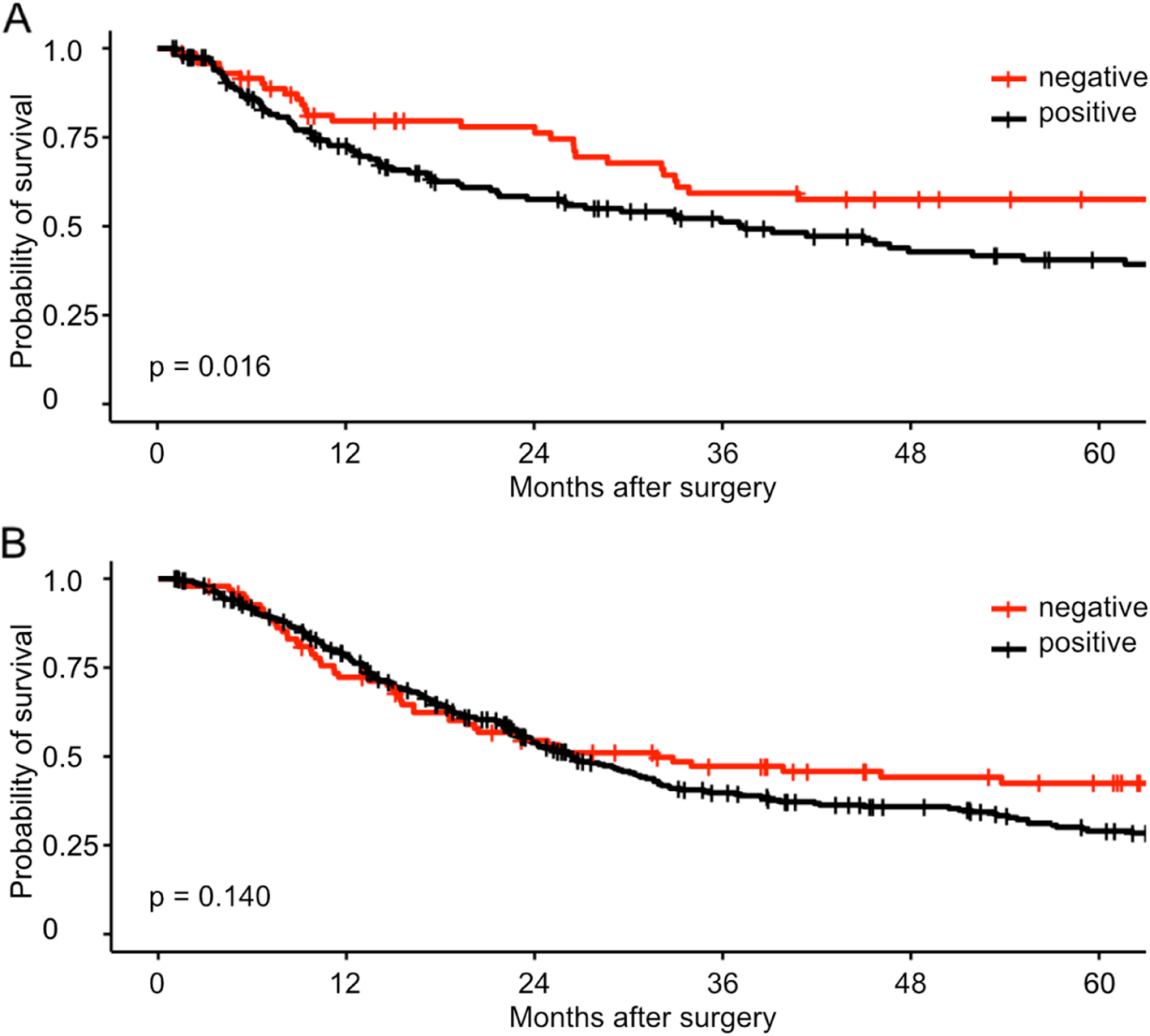
Analyses of overall survival depending on negative or positive expression of NANOG for the subgroups of patients (**A**) primary surgery (negative: *n* = 72, median Follow-Up = 33.49 months, positive: *n* = 156, median Follow-Up = 17.45 months, *p *= 0.016) or (**B**) after neoadjuvant therapy (negative: *n* = 96, median Follow-Up = 23.20 months, positive: *n* = 336, median Follow-Up = 22.16 months, *p* = 0.140)

We then separately analyzed the influence of NANOG expression in these two subgroups. Positive NANOG expression showed a significant correlation with worse survival in patients who were operated primarily (*p* = 0.016, Fig. 2 A). On the contrary, no correlation of NANOG expression with patient survival could be detected in the subgroup of patients who underwent a neoadjuvant therapy (*p* = 0.140, Fig. 2 B). To gain a better understanding of the role of NANOG expression in patients with different neoadjuvant treatments, we conducted survival analyses in these subgroups. Patients treated with FLOT did not show any survival differences between NANOG negative and positive subgroups (*p* = 0.610). Moreover, NANOG-positive patients with tumors previously treated according to the CROSS trial exhibited a trend towards worse survival (*p* = 0.060).

Subsequent sub-analyses detected positive NANOG expression, higher age, higher pT-, and pN-stage as independent risk factors for worse patient survival (NANOG expression: HR = 1.72, 95% CI = 1.10–2.67, *p* = 0.013; age: HR = 1.04, 95% CI = 1.02–1.06, *p* =  < 0.001; pT 4 vs 1: HR = 5.02, 95% CI = 1.72–14.6, *p* < 0.001; pN 3 vs 0: HR = 5.18, 95% CI = 2.83–9.46, *p* < 0.001, Table 3).

**Table 3:** Multivariate cox regression of the patients with primary surgery. Bold print marks p-values below 0.05

Characteristic	Borders	Hazard Ratio	95% confidence interval	*p*—value
**Sex**	male vs female	1.64	0.87—3.09	0.110
**Age**		1.04	1.02—1.06	** < 0.001**
**pT**				** < 0.001**
	2 vs 1	1.34	0.63—2.85	
	3 vs 1	2.69	1.49—4.87	
	4 vs 1	5.02	1.72—14.6	
**pN**				** < 0.001**
	1 vs 0	2.68	1.58—4.54	
	2 vs 0	2.25	1.12—4.54	
	3 vs 0	5.18	2.83—9.46	
**NANOG**	positive vs negative	1.72	1.10—2.67	**0.013**

The results of the multivariate cox regression analyses for the neoadjuvant treated patients are depicted in Supp. Table 4. Here, only the pN-status could be defined as an independent risk factor for worse survival.

Since it is known, that TP53 influences NANOG expression, we conducted immunohistochemical TP53 stainings in 256 patients of our Nanog patient cohort. Following this, we performed multivariate cox regression analysis for this subcohort. Interestingly, neither Nanog nor TP53 proves to be a significant risk factor for patients' survival in this subcohort (Nanog: HR = 1.24, 95% CI = 0.85–1.83, *p* = 0.300; TP53: HR = 1.31, 95% CI = 0.93–1.85, *p* = 0.120). However, neoadjuvant therapy, age, and pN are significant independent risk factors for worse survival (neoadjuvant therapy: HR = 1.55, 95% CI = 1.04–2.32, *p* = 0.027; age: HR = 1.02, 95% CI = 1.00–1.04, *p* = 0.026; pN 3 vs 0: HR = 3.22, 95% CI = 1.96–5.29, *p* < 0.001).

**Fig. 3:**
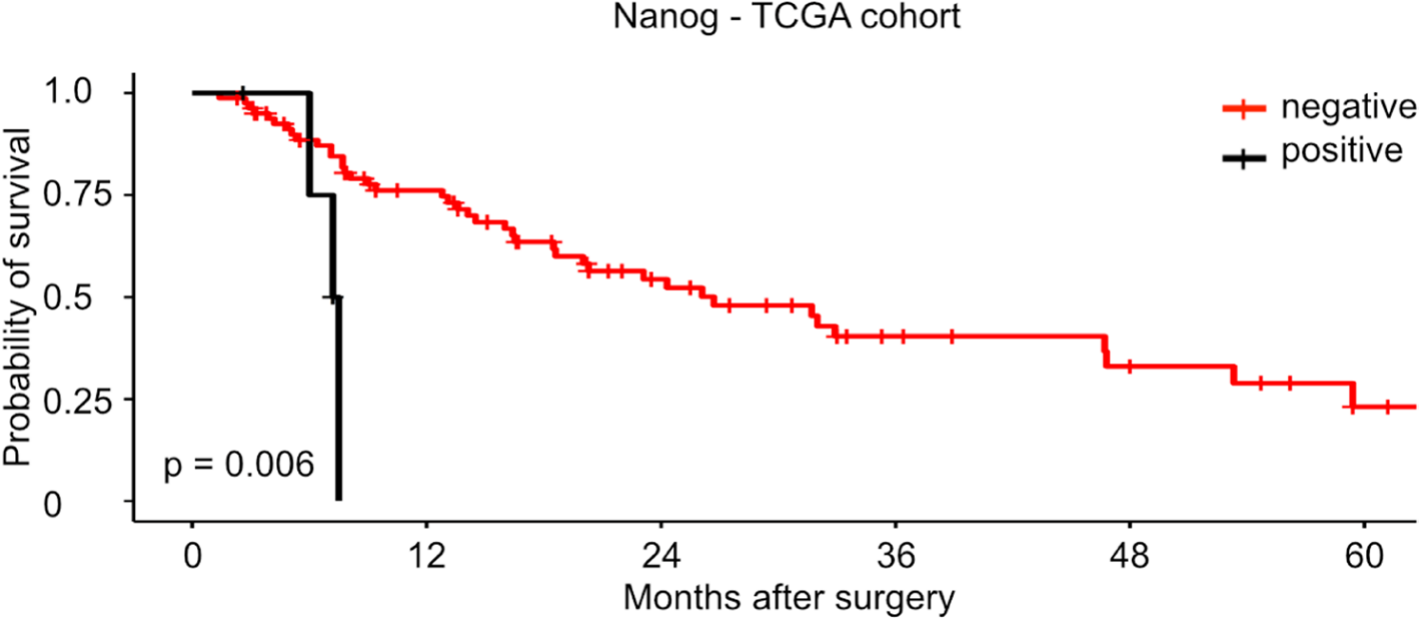
Kaplan Meier curve of the TCGA cohort divided in patients with NANOG expression and without NANOG expression (n(negative) = 83, n(positive) = 5, *p* = 0.006). TCGA: The Cancer Genome Atlas

To verify our results in an independent patient cohort, we analyzed the RNA-Sequencing data of the TCGA cohort. All included patients in the TCGA cohort were treated with primary surgery solely. When dichotomized 5 tumors (5.7%) had a NANOG expression of TPM > 5.0, while 83 (94.3%) tumors displayed NANOG mRNA expression levels below this threshold. The NANOG expression was not significantly associated with patients’ age, sex, tumor stage, lymph node stage, AJCC stage or the presence of distant metastases (Fisher’s exact test and Spearman correlation test, all tests *p* > 0.05, Supp. Table 5). Patients with tumors which expressed NANOG had a significantly shorter overall survival (OS) than patients with esophageal adenocarcinomas without NANOG expression (median OS: 7.3 months vs. 26.7 months; *p* = 0.006, Fig. 3).

In univariate analysis, positive NANOG expression was a significant adverse prognostic factor in the TCGA cohort (HR = 4.98, 95% CI = 1.398—17.75, *p* = 0.013). Other significant factors in univariate analysis were lymph node stage and distant metastasis (*p* = 0.005, *p* = 0.001). In multivariate analysis, NANOG expression remained a significant, independent prognostic factor for worse patient survival (*p* = 0.001).

In summary, our data show that NANOG expression correlates with poor patient survival in the total population and in the subgroup of patients who were operated primarily. Additionally, positive NANOG expression is an independent risk factor for worse survival in these groups.

## Discussion

In this study, 660 esophageal adenocarcinoma samples were evaluated regarding their NANOG expression and its possible influence on clinicopathologic characteristics and overall survival. Higher age, higher pN-, pT-, UICC-stage, and positive NANOG-expression were factors for worse patients’ survival in univariate cox regression analyses. Furthermore, we identified NANOG expression as an independent risk factor for worse overall survival. We could confirm this in an independent cohort. However, the TCGA databank provided only information on 88 patients. Moreover, only 5 patients had a positive NANOG expression. Further confirmation in independent cohorts is needed in future projects. The identified effect was previously described in—among others—oral squamous cell cancer as well as non-small cell lung cancer [14, 15]. Sun et al. were able to establish a risk score in esophageal squamous cell carcinoma. A higher risk score, which is determined based on immunohistochemical stainings of PPARG, MDM2, and NANOG, is correlated with worse overall survival [16]. Immunohistochemical analyses of NANOG expression in 115 patients suffering from esophageal squamous cell carcinomas and 19 patients suffering from adenocarcinomas showed that positive NANOG expression correlated significantly with worse survival [17]. Interestingly, this effect could be confirmed in patients with neoadjuvant therapy, but not in patients who underwent primary surgery [17]. We found a significant correlation between positive NANOG expression and decreased survival in patients who underwent primary surgery, while this association was not observed in patients who received neoadjuvant therapy. These differences could be explained by the small sample size used by Narusaka et al. Furthermore, Narusaka et al. included mainly squamous cell carcinomas (87%) in contrast to our study cohort, which included only adenocarcinomas. In line with our results, Hwang et al. could show no survival differences depending on the NANOG expression status in a patient cohort of 41 neoadjuvant treated patients with esophageal squamous cell carcinoma [18]. An alternative explanation could be that the patients were treated with different neoadjuvant therapy regimes due to possible national differences in guidelines as well as in recruitment periods. Additionally, we hypothesize, that the missing prognostic effect of NANOG in the neoadjuvant subgroup could be explained due to a high proportion of minor responders in our tissue microarray, since full responders and hypothetically NANOG-negative could not be included due to the lack of vital tumor tissue that would have necessary to assess for NANOG expression status [17]. Patients with a full response are known to have better survival [19, 20]. It is suspected that NANOG-positive tumors may exhibit a chemoresistant phenotype. To address this limitation and include potential pathological complete responders, future studies could assess NANOG expression in primary biopsies before any therapy is administered. If the assumption, that pathological complete responders after neoadjuvant therapy are mainly NANOG negative proves to be right, a higher NANOG expression could advocate for adjuvant therapy not only in the primarily operated patients but also in the neoadjuvant treated patients. This would increase the clinical value of NANOG since the majority of patients receives multimodal treatment regimes due to recent guidelines [21]. Additionally, NANOG is modulated by a complex regulatory system involving the widely known TP53 protein [8]. Therefore, we performed TP53 stainings in a subcohort and conducted analyses to correct our survival analyses for interdependencies. Interestingly, neither TP53 nor NANOG showed to be an independent risk factor in these analyses. The lack of significance could be explained by either the smaller subcohort or the influence of TP53. Consequently, further investigations regarding the influence between TP53 and NANOG are needed.

Furthermore, neoadjuvant therapy was a factor for worse survival. However, it should be noted that patients receiving neoadjuvant therapy are typically diagnosed with a clinical higher tumor stage. Although neoadjuvant therapy has been shown to provide a survival benefit for patients within the same tumor stage, their survival is still worse compared to patients with an earlier tumor stage. This hypothesis is supported by the fact that neoadjuvant therapy was not found to be a significant factor for patient survival in the multivariate cox regression analysis of the total cohort.

We could showcase that in our patient cohort significantly more NANOG-positive samples were detected in patients after neoadjuvant therapy. Neoadjuvant therapy is recommended for patients with higher pre-therapeutic tumor characteristics according to guidelines [21, 22]. We postulate, that NANOG-positive tumors tend to have a higher tumor stage. Moreover, neoadjuvant therapy leads to a hypoxic milieu in solid tumors [23]. In fact, elevated NANOG levels in cancer cells are known to be caused by hypoxia [24, 25]. However, further research is needed to evaluate the impact of neoadjuvant treatment on NANOG expression in esophageal adenocarcinoma—especially dependent on the difference between chemotherapy versus chemoradiotherapy [26, 27]. The administered neoadjuvant treatment regime was unknown in a part of our study cohort. Therefore, our above-mentioned analyses regarding NANOG expression in different neoadjuvant treated patients should be confirmed in future projects.

Analyses of human tissue microarrays with pancreatic cancer and non-cancerous tissues could detect NANOG expression in metaplastic ducts, suggesting that NANOG is already involved in the early stages of carcinogenesis [28]. NANOG inhibition could result in suppression of cell proliferation in colorectal cancer cell lines [29]. This could designate NANOG as a potential treatment target. Indeed, new therapy options have already been developed in acute myelogenous leukemia [30]. Additionally, NANOG expression could lead to altered treatment administration of already established drugs. In patients with skin melanoma NANOG related suppression of the immune response results in an inadequate response to anti-PD-1-therapy [31]. This finding is particularly relevant for esophageal cancer, as Nivolumab is emerging as adjuvant therapy for patients who have undergone neoadjuvant chemoradiotherapy according to the CROSS protocol [32].

Although this study is limited by its retrospective design, the large size of our cohort (660 patients) allowed us to provide comprehensive insights into the role of NANOG expression in esophageal cancer. Mechanistic experiments should elucidate the pathomechanism of NANOG as well as the stem-like biological properties and their influence on (radio-) chemoresistance as well as patient survival in esophageal adenocarcinoma. Assessing NANOG expression in primary biopsies prior to neoadjuvant therapy could provide valuable information for clinicians. This could lead to the modification or intensification of neoadjuvant therapy for tumors that are chemoresistant to current systemic treatment options and have a high NANOG expression. This may significantly improve patients' survival and alter the current treatment algorithm. However, further prospective studies are needed to confirm that NANOG-positive tumors represent a high-risk subgroup with low response to neoadjuvant treatment in esophageal adenocarcinoma.

## Conclusions

Patients with NANOG expressing esophageal adenocarcinoma are accumulated in the neoadjuvant treated subgroup and showed to have a significantly worse overall survival in our study cohort. Therefore, we describe a NANOG-positive subgroup, which shows a worse survival under the current standard of care. We hypothesize that these patients could benefit after further mechanistic and prospective studies of intensified multimodal therapy.

## Supplementary Information

**Additional file 1.** 

## Data Availability

The datasets generated and analyzed during the current study are available from the corresponding author upon reasonable request.

## Abbreviation

AJCC: American Joint Committee on Cancer
CI: Confidence interval
EAC: Esophageal adenocarcinoma
et al.: Et alia (and others)
GDAC: Genome Data Analysis Center
HR: Hazard Ratio
MDM 2: Mouse double minute 2 homolog
Mm: Millimeter
OS: Overall survival
PD-1: Programmed cell death protein 1
PPARG: Peroxisome proliferator- activated receptor gamma
RNA: Ribonucleic acid
STAT3: Signal transducer and activator of transcription 3
TCGA: The Cancer Genome Atlas
TMA: Tissue microarray
TPM: Transcripts per Million
UICC: Union for International Cancer Control
Vs: Versus
