# Supplementary material for: High NANOG expression correlates with worse patients’ survival in esophageal adenocarcinoma
Source: BMC Cancer. 2023 Jul 17;23:669. doi: 10.1186/s12885-023-11146-0 (PMC10351130; doi:10.1186/s12885-023-11146-0)
Supplement: Supplementary file 1 — Additional file 1. [file 12885_2023_11146_MOESM1_ESM.docx]

**Supp. Figure 1**

Representative images (200X) of the different Nanog expression patterns: (A) negative, and (B) positive.

**
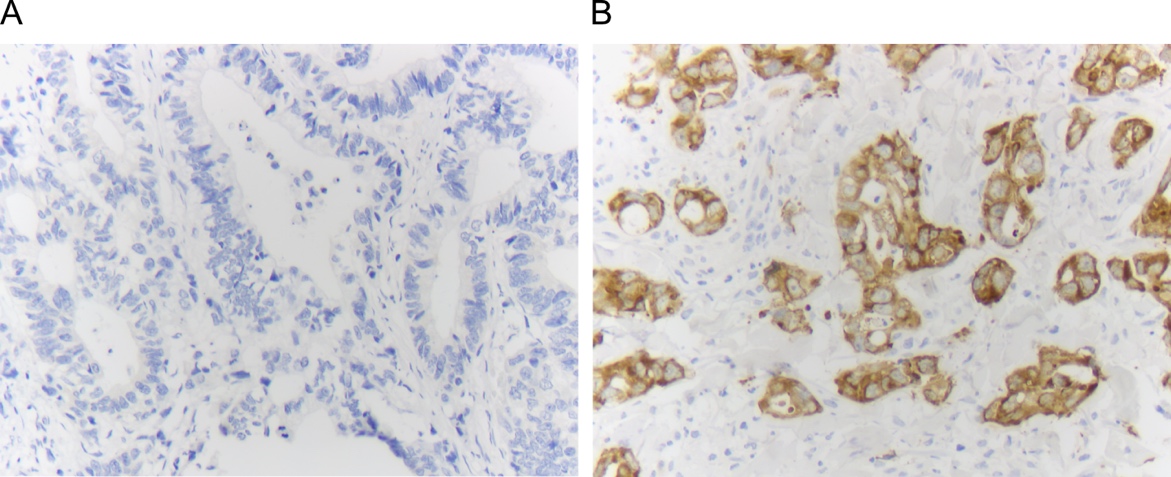
**

**Supp. Table 1**

Detailed antibody information.

| **Antibody** | **Manufacturer** | **Clone** | **Dilution** | **Pretreatment** | **Control** | **Order number** |
| --- | --- | --- | --- | --- | --- | --- |
| Nanog | Cellsignaling | D73G4 | 1:100 | EDTA | Seminoma | 4903S |

**Supp. Table 2**

Univariate cox regression of the total population. UICC: Union for International Cancer Control. Bold print marks p-values below 0.05.

| **Characteristic** | **Borders** | **Hazard Ratio** | **95 % confidence interval** | **p - value** |
| --- | --- | --- | --- | --- |
| **Sex** | male vs female | 1.35 | 0.98 - 1.87 | 0.058 |
|  |  |  |  |  |
| **Age** |  | 1.02 | 1.01 - 1.03 | **<0.001** |
|  |  |  |  |  |
| **Neoadjuvant therapy** | yes vs no | 1.37 | 1.09 - 1.71 | **0.005** |
|  |  |  |  |  |
| **pT** |  |  |  | **< 0.001** |
|  | 2 vs 1 | 2.47 | 1.64 - 3.73 |  |
|  | 3 vs 1 | 3.06 | 2.16 - 4.33 |  |
|  | 4 vs 1 | 5.05 | 2.66 - 9.57 |  |
|  |  |  |  |  |
| **pN** |  |  |  | **< 0.001** |
|  | 1 vs 0 | 2.23 | 1.71 - 2.92 |  |
|  | 2 vs 0 | 2.82 | 2.06 - 3.85 |  |
|  | 3 vs 0 | 4.94 | 3.68 - 6.65 |  |
|  |  |  |  |  |
| **UICC** |  |  |  | **<0.001** |
|  | 2 vs 1 | 2.61 | 1.51 - 4.51 |  |
|  | 3 vs 1 | 3.44 | 2.16 - 5.50 |  |
|  | 4 vs 1 | 6.96 | 4.34 - 11.2 |  |
|  |  |  |  |  |
| **Nanog** | positive vs negative | 1.46 | 1.14 - 1.86 | **0.002** |

**Supp. Table 3**

Patients’ characteristics of patients with neoadjuvant therapy or primary surgery. UICC: Union for International Cancer Control. Bold print marks p-values below 0.05.

| **Characteristic** | **Neoadjuvant therapy** | | | | **Primary surgery** | | | |
| --- | --- | --- | --- | --- | --- | --- | --- | --- |
|  | **Overall** | **NANOG** | **NANOG** | **p - value** | **Overall** | **NANOG** | **NANOG** | **p - value** |
|  |  | **negative** | **positive** |  |  | **negative** | **positive** |  |
|  | **n (%)** | **n (%)** | **n (%)** |  | **n (%)** | **n (%)** | **n (%)** |  |
| **No. of patients** | 432 (100) | 96 (100) | 336 (100) |  | 228 (100) | 72 (100) | 156 (100) |  |
| **Sex** |  |  |  | 0.200 |  |  |  | 0.080 |
| Male | 379 (88) | 88 (92) | 291 (87) |  | 197 (86) | 58 (81) | 139 (89) |  |
| Female | 53 (12) | 8 (8.3) | 45 (13) |  | 31 (14) | 14 (19) | 17 (11) |  |
| **Median Age (years)** | 61 | 63 | 61 | 0.300 | 68 | 70 | 68 | 0.400 |
| **(range)** | (54-68) | (56-68) | (54-68) |  | (58-75) | (58-76) | (57-75) |  |
| **Neoadjuvant therapy** |  |  |  | 0.832 |  |  |  | - |
| CROSS | 142 (33) | 26 (27) | 116 (35) |  | 0 (0) | 0 (0) | 0 (0) |  |
| FLOT | 77 (18) | 15 (16) | 62 (18) |  | 0 (0) | 0 (0) | 0 (0) |  |
| not specified | 213 (49) | 55 (57) | 158 (47) |  | 0 (0) | 0 (0) | 0 (0) |  |
| **NANOG** |  |  |  |  |  |  |  | **0.009** |
| negative | 96 (22) | 96 (100) | 0 (0) |  | 72 (32) | 72 (100) | 0 (0) |  |
| positive | 336 (78) | 0 (0) | 336 (100) |  | 156 (69) | 0 (0) | 156 (100) |  |
| **pT** |  |  |  | 0.400 |  |  |  | 0.600 |
| 1 | 39 (9) | 11 (11) | 28 (8) |  | 68 (30) | 26 (36) | 42 (27) |  |
| 2 | 74 (17) | 15 (16) | 59 (18) |  | 33 (14) | 9 (12) | 24 (15) |  |
| 3 | 305 (71) | 69 (72) | 236 (70) |  | 119 (52) | 35 (49) | 84 (54) |  |
| 4 | 14 (3) | 1 (1) | 13 (4) |  | 8 (4) | 2 (3) | 6 (4) |  |
| **pN** |  |  |  | 0.300 |  |  |  | 0.300 |
| 0 | 150 (35) | 41 (43) | 109 (32) |  | 96 (42) | 35 (49) | 61 (39) |  |
| 1 | 133 (30) | 26 (27) | 107 (32) |  | 68 (30) | 22 (31) | 46 (29) |  |
| 2 | 77 (18) | 15 (16) | 62 (19) |  | 26 (11) | 7 (10) | 19 (12) |  |
| 3 | 72 (17) | 14 (15) | 58 (17) |  | 38 (17) | 8 (11) | 30 (20) |  |
| **UICC** |  |  |  | 0.700 |  |  |  | 0.400 |
| I | 24 (6) | 7 (7) | 17 (5) |  | 53 (23) | 20 (28) | 33 (21) |  |
| II | 43 (10) | 10 (11) | 33 (10) |  | 27 (12) | 10 (14) | 17 (11) |  |
| III | 216 (50) | 50 (52) | 166 (49) |  | 84 (37) | 27 (37) | 57 (38) |  |
| IV | 149 (34) | 29 (30) | 120 (36) |  | 64 (28) | 15 (21) | 49 (31) |  |

**Supp. Table 4**

Multivariate cox regression of the neoadjuvant treated patients. Bold print marks p-values below 0.05.

| **Characteristic** | **Borders** | **Hazard Ratio** | **95 % confidence interval** | **p - value** |
| --- | --- | --- | --- | --- |
| **Sex** | male vs female | 1.30 | 0.88 - 1.93 | 0.200 |
|  |  |  |  |  |
| **Age** |  | 1.01 | 1.00 - 1.02 | 0.110 |
|  |  |  |  |  |
| **pT** |  |  |  | 0.700 |
|  | 2 vs 1 | 1.34 | 0.79 - 2.30 |  |
|  | 3 vs 1 | 1.29 | 0.80 - 2.09 |  |
|  | 4 vs 1 | 1.17 | 0.48 - 2.88 |  |
|  |  |  |  |  |
| **pN** |  |  |  | **<0.001** |
|  | 1 vs 0 | 1.71 | 1.24 - 2.37 |  |
|  | 2 vs 0 | 2.12 | 1.47 - 2.37 |  |
|  | 3 vs 0 | 3.36 | 2.34 - 4.83 |  |
|  |  |  |  |  |
| **Nanog** | positive vs negative | 1.25 | 0.92 - 1.69 | 0.140 |

**Supp. Table 5**

Patients’ characteristics of the total TCGA cohort and divided by the NANOG expression. AJCC: American Joint Committee on Cancer, TPM: Transcripts per Million.

| **Characteristic** | **Total**  **n (%)** | ***NANOG***  **TPM < 5.0**  **n (%)** | ***NANOG***  **TPM > 5.0**  **n (%)** | **p - value** |
| --- | --- | --- | --- | --- |
| **No. of patients** | 88 (100) | 83 (100) | 5 (100) |  |
| **Sex** |  |  |  | 0.135 |
| Male | 76 (86) | 73 (88) | 3 (60) |  |
| Female | 12 (14) | 10 (12) | 2 (40) |  |
| **Median Age (years)** | 69 | 70 | 60 |  |
| **(range)** | (28 – 86) | (28 – 86) | (56-74) |  |
| ***NANOG*** |  |  |  |  |
| TPM < 5.0 | 83 (94) | 83 (100) | 0 |  |
| TPM > 5.0 | 5 (6) | 0 | 5 (100) |  |
| **pT** |  |  |  | 0.394 |
| 1 | 22 (25) | 21 (25) | 1 (20) |  |
| 2 | 11 (13) | 9 (11) | 2 (40) |  |
| 3 | 38 (43) | 36 (43) | 2 (40) |  |
| 4 | 1 (1) | 1 (1) | 0 (0) |  |
| Unknown | 16 (18) | 16 (19) | 0 (0) |  |
| **pN** |  |  |  | 1.0 |
| 0 | 21 (24) | 20 (24) | 1 (20) |  |
| ≥ 1 | 51 (58) | 47 (57) | 4 (80) |  |
| Unknown | 16 (18) | 16 (19) | 0 (0) |  |
| **AJCC grade** |  |  |  | 1.0 |
| 1 | 11 (13) | 10 (12) | 1 (20) |  |
| 2 | 23 (26) | 21 (25) | 2 (40) |  |
| 3 | 29 (33) | 27 (33) | 2 (40) |  |
| 4 | 5 (6) | 5 (6) | 0 (0) |  |
| Unknown | 20 (23) | 20 (24) | 0 (0) |  |
